# Teenage pregnancy and experience of physical violence among women aged 15-19 years in five African countries: Analysis of complex survey data

**DOI:** 10.1371/journal.pone.0241348

**Published:** 2020-10-27

**Authors:** John Tetteh, Benjamin D. Nuertey, Duah Dwomoh, Emilia Asuquo Udofia, Sheriff Mohammed, Evelyn Adjei-Mensah, Alfred Edwin Yawson

**Affiliations:** 1 Department of Community Health, University of Ghana Medical School, College of Health Sciences, University of Ghana, Accra, Ghana; 2 Department of Biostatistics, School of Public Health, College of Health Sciences, University of Ghana, Accra, Ghana; 3 National Cardiothoracic Centre, Korle-Bu Teaching Hospital, Accra, Ghana; University of Sao Paulo Medical School, BRAZIL

## Abstract

**Background:**

Pregnant teenage women are prime targets of violence against women perpetrated by intimate partners, family members, and miscreants in their neighborhoods. This study estimated the prevalence of Teenage pregnancy (TP) and Physical Violence (PV) and further assessed the relationship between TP and PV in five Low-and-Middle-Income Countries (LMICs).

**Methods:**

The study was conducted among five LIMCs (Burkina Faso, Kenya, Malawi, Nigeria, and Tanzania) using data from the most recent Demographic and Health Surveys conducted in these countries. Modified Poisson with the robust standard error was used to quantify the association between TP and PV. All analyses adjusted for the complex survey design structure (clustering, weighting, and stratification).

**Results:**

The analysis involved a total of 26055 adolescent women aged 15–19 years across the five countries. The overall prevalence of TP was 25.4% (95%CI = 24.4–26.4) with the highest prevalence occurring among Malawians [29.0% (95%CI = 27.4–30.7)]. Meanwhile, the prevalence of TP among older adolescents (18–19 years) was approximately two-thirds significantly higher compared with young adolescents [aPR(95%CI) = 1.60[1.49–1.71)]. The prevalence of PV among teenagers across the five countries was 24.2% (95%CI = 22.3–26.2). The highest prevalence of PV was recorded among Nigerian adolescent women [31.8% (95%CI = 28.5–35.3)]. The prevalence of PV among adolescent women who were pregnant was approximately 5-folds significant compared to those who were not pregnant (adjusted prevalence ratio; aPR = 4.70; 95% CI: 3.86–5.73; p<0.0001).

**Conclusion:**

There was a high prevalence of pregnancy among older teenagers aged 18–19 years. Close to a quarter of teenage women ever experienced physical violence. Pregnant teenage women ever experience of physical violence was very high compared to non-pregnant peers. Intervention should target PV and TP by adopting a gender-sensitive approach to eliminate physical violence, particularly among teenagers to prevent TP.

## Introduction

Every year, an estimated 21 million teenage girls aged 15–19 years become pregnant [1], of which 16 million give birth. Birth among older teenagers aged 15–19 years accounts for 11% of worldwide births, 95% of which occur in low and middle-income countries (LMIC) [2,3]. Sub-Saharan Africa has the highest teenage pregnancy and delivery rates in the world and the rates are still increasing [4]. In some parts of Africa, more than 10% of adolescent girls become mothers before the age of 16 years [5]. Teenage birth rates in Africa are highest with 115 births per 1000 women, with urban-rural disparities where there are up to three times more teenage pregnancies in rural populations than urban populations [6].

Increased rate of Teenage pregnancy (TP) rates in Africa have been influenced over the decades by reasons such as progressively lower age of menarche, initiation of first sexual activity at a progressively lower age, and low contraceptive use rates among teenagers [7]. TP is viewed as a complex socioeconomic and medical problem in itself, with immediate and long-term adverse effects. Each year, an estimated 3.9 million unsafe abortions are carried out among girls aged 15–19 years, contributing to maternal mortality and other immediate and long term health problems [1]. Among those who opt to give birth, the complications are equally worse.

Many studies from almost every part of the globe consistently and convincingly report adverse obstetric, perinatal, medical, and socioeconomic outcomes among TP women and their children. Pregnant teenage women are at an increased risk of adverse obstetric complications such as anaemia in pregnancy [8], pregnancy-induced hypertension, and associated hypertensive disorders of pregnancy [9–11]. Others include preterm labour, cephalopelvic disproportion, higher incidence of operative delivery [4,12,13], post-partum hemorrhage, and associated blood transfusion. Major perineal lacerations and need for episiotomy [14], obstetric fistula [15], and postnatal depression [10] also occur more frequently in teenage pregnancies. It is often cited that teenagers who give birth aged 15–19 years are more than twice as likely to die as those aged 20 years and above [5].

The foetus, neonates, and infants of teenage mothers are at an increased risk of preterm delivery, low birth weight, small for gestational age, low APGAR (Appearance, Pulse, Grimace, Activity, and Respiration) scores [2,7,16–19], death in the perinatal, neonatal and infant periods [4,20–23], increased Neonatal Intensive Care Unit (NICU) admissions [14,24–26], higher neonatal morbidities [27,28], congenital malformations [10,24,25], developmental delays and behavioural disorders [29].

Other health and socio-economic complications include a higher incidence of HIV/AIDS among unmarried pregnant teenagers, termination of academic pursuits [4,30], teenage mothers struggle due to lack of preparedness for childbearing, negative public attitude directed towards adolescent pregnant women [31], mental health problems, and socioeconomic disadvantage [32]. Other social problems include single parenthood, with long term effects of increased risk of child abuse, child neglect, maternal suicide, and repeated suicidal attempts [19]. Additionally, offspring of teenage pregnancies are more likely to end up with teenage pregnancies, perpetuating the cycle of teenage pregnancy. The adverse effects of teenage pregnancies are often potentiated due to certain behavioural tendencies that make teenage pregnant women hide the pregnancy and they are more likely to remain un-booked for antenatal services even up to the time of delivery [33,34].

In developing countries, it is estimated that as high as 90% of teenage pregnancy occurs within marriage bringing to bear the contribution of early marriages [35]. The prevalence of intimate partner violence (IPV) during pregnancy has been reported as 40% from a review of facility-based studies in Africa [1,36]. The prevalence of IPV among pregnant teenage women aged 15–19 years in Africa is not known but believed to be relatively higher than among older age groups. Elsewhere outside Africa, studies have found out that, women aged less than 20 years had 4.3 times of experiencing violence during pregnancy compared to women aged 30 years and above [37]. Physical violence among pregnant women has been associated with miscarriage, stillbirth, premature labour and birth, foetal injury, and low birth weight [36,38]. Pregnant teenagers were significantly more likely to have experienced forced sexual initiation, experienced physical violence in the form of beating more often [39] and other physical, sexual, or domestic violence in attempts to meet the demands of pregnancy and childbearing. This abuse in pregnancy is significantly associated with adverse obstetric outcomes among teenage pregnant women [40]. The prevalence of nationwide estimates of TP in Africa and rates of experienced physical abuse among pregnant teenagers aged 15–19 years and its predictive factors are significantly lacking in extant literature.

Even when available, previous studies in the area of teenage pregnancy suffered from inherent design limitations such as small sample size, inadequate confounder information, unresolved bias, and studies limited to healthcare facilities. Even the population studies fail to cover wide geographical areas making findings from such studies to have limited generalisability and power to effect change. The main objective of this population-based study was to determine the association between TP and PV among adolescent women aged 15–19 years living in five low- and middle-income countries in Africa. The study uses recent demographic and health survey data from five African countries.

## Methods

### Study description

The study was conducted among five low-and-middle-Income countries namely Burkina Faso, Kenya, Malawi, Nigeria, and Tanzania. Demographic and Health Survey (DHS) data for these countries were pooled for analysis and results presented to inform targeted intervention. DHS data was obtained for Burkina Faso (2010), Kenya (2014), Malawi, and Tanzania (2015/2016), and Nigeria (2018). These countries recent DHS module assessed domestic physical violence which other counties did not. Our main objective was to assess the association between ever experience physical violence and teenage pregnancy. This is deemed appropriate for including these counties.

### Study design

The DHS surveys collect information on a wide range of variables for a sample of countries that participate in the survey. Globally, DHS has earned a worldwide reputation for collecting nationally representative data on different aspects of health indicators including fertility issues among men and women in childbearing years, family planning, maternal and child health, gender, HIV/AIDS, malaria, and nutrition, domestic violence since 1984 [41]. The Monitoring and Evaluation to Assess and Use Results Demographic and Health Surveys (MEASURE DHS) funded by the U.S. Agency for International Development (USAID) is responsible for providing technical assistance to all DHS surveys in over 90 countries to enhance global understanding of the health and population in developing countries [42]. DHS collects data using a cross-sectional design and employs a two-stage sampling of all geographical regions in a country based on enumeration areas (EAs). Details about the study design and procedures for data collection have been published elsewhere [43].

### Study participants

DHS collects information on all aspects of health including household (HH), child, men, and women individual records. However, the present study focused on the women survey (women in their reproductive age, 15–49 years) and their respective HH records. To answer the study objectives, the present study focuses on older teenagers aged 15–19 years. The data for the individual women were merged with their corresponding households to obtain complete data. The sample for all five countries is 26,055. The sample was stratified across the five countries as follows: Burkina Faso (3349), Kenya (6,078), Malawi (5,273), Nigeria (8423), and Tanzania (2,932). Missing responses in some variables are clearly defined in S1 Table.

### Outcome measures

In this study, two outcome variables were considered; namely, TP and PV. We assessed the association between TP and ever experienced domestic PV. TP has been identified as a public health issue that can cause severe health problems for a mother and her born or unborn child [1]. DHS defined TP as a percentage of reproductive teenage girls who are mothers, pregnant with their first child, and have begun childbearing [44]. In the present study, TP was estimated based on:

1. Women aged 15–19 years and have had a live birth.
2. Women aged 15–19 years who were pregnant with their first child.
3. Women aged 15–19 years who have begun childbearing.

After generating the index variable for TP, it was categorized as a dummy variable (Yes = 1 or No = 0). A detailed process and variable definition can be found in S1 Table.

Ever experience of PV was as assessed by DHS; where women aged 15–49 years were asked whether they have ever experienced physical violence during the past 12 months (often or sometimes) preceding the survey, however, our research only considered those aged 15–19 years. DHS collected information on women who have experienced any form of physical violence scaled on 12 items. The items were about; any husband/partner ever if ever-married; anyone other than any husband/partner since they were age 15 years; and anyone during any pregnancy, if ever pregnant. The 12-item scale physical violence module was naturally coded as; never, sometimes, or often. Based on the objective of our study, an artificial dummy variable was generated for all the 12 items by recoding into; as 0 “Never experienced” or 1 “Ever experienced (sometime and often)”. An index variable from the raw scores (ranging from 0–12) was generated and further reclassified into a modified binary primary outcome variable as 0 “Never experienced” or 1 “Ever experienced (any of the 12-item)”. For reliability and internal consistency, the Jann Stata module was used to compute Cronbach’s alpha for weighted data due to DHS design [45]. The overall test of reliability for physical violence was very high and of good quality (α = 0.76). The items all tapped into the same concept of measurement (see S2 Table).

### Covariate

#### Household (HH) and community characteristic

Age of HH head (<30, 30–39, 40–49, 50–59, 60–59 and 70+), sex of HH head (Male or Female), HH has Telephone (No or Yes), HH wealth index (Poorest, poorer, middle, richer and richest), the number of HH members (<4, 4–5, 6–8 and 9+), HH has electricity (No or Yes), HH has a radio(No or Yes), HH has color television (No or Yes), where HH food is prepared (In the household, separate household and others), HH has a mobile phone (No or Yes), HH has a watch (No or Yes), HH own land for agriculture (No or Yes), HH number of animals own (None or 1+animal), relationship structure (No+1 adult; two adults, opposite sex; two adults, same sex; three+ related adults and unrelated adults) and pace of residence (Urban or Rural). Detailed variable definition, type of variable, measurement, and scale of measurement used in this study has been clearly defined in S1 Table.

#### Individual characteristics

Age of participant (15–17 and 19–19), educational level (None, primary, secondary or higher), marital status (Never married, married, divorced/widowed), knowledge on pregnancy (No knowledge and have knowledge), knows modern contraceptive (Knows no method and knows method), family planning awareness (aware and aware), currently abstaining (No or Yes) and currently working (No or Yes).

Based on Strengthening the Reporting of Observational Studies in Epidemiology (STROBE) recommendation for cross-sectional study design, implementation, and reporting, missing responses were strictly excluded in our analysis [46]. Detailed variable definition, type of variable, measurement, and scale of measurement used in this study has been clearly defined in S1 Table.

### Data analysis

This study adjusted for the design of DHS since it is a complex survey. In preventing wrong estimations, adjusting for the participants’ weighting, stratification, and clustering in a complex survey dataset is a key issue to consider during data analysis. DHS adopted a multistage cluster survey design and due to the complex design of DHS, this study adjusted for the primary sampling units, stratification, and the sampling weights to reduce bias and to improve data analysis in all estimates. Since the data used were pooled from five countries at different time points, the individual women and domestic violence weights were normalized due to different population sizes. The normalized sampling weight was calculated by multiplying the raw weight by the overall

Sampling fraction:

$$w_{N}=w_{15-49}*\frac{n_{15-49}}{N_{15-49}}$$

Where *w*_15–49_ are the women aged 15–49 individual sampling weight estimated by DHS, *n*_15–49_ is the number of women aged 15–49 years interviewed in the country-specific survey and *N*_15–49_ is the total number of women aged 15–49 years in the country at the time of the survey. The same principle for normalizing individual sampling weights was applied to domestic violence sampling weights. The total number of women aged 15–49 interviewed in the survey year was obtained from the DHS datasets, while the total number of women aged 15–49 years in the country at the time of the survey was obtained from the United Nations (UN) Department of Economic and Social Affairs, Population Dynamics [47]. The population information from DHS and the UN can be found in S3 Table.

Three approaches to data analysis were carried out. First, bivariate descriptive statistics were generated with Rao-Scott chi-square for testing the independence of covariate variables among the various countries and how different they are. Before performing inferential analysis, we analyzed to assess multicollinearity among the variables considered by adopting the variance inflation factor (VIF) and pairwise correlation. The analysis showed no suspected multicollinearity (overall mean VIF< 1.34) and also, none of the individual variables’ pairwise correlation coefficient is ≥0.8.

Secondly, factors influencing TP were determined by using the modified Poison regression model. Normalized domestic violence weight was considered for the quantification of association between TP and ever experience domestic violence adjusting for significant factors influencing TP. Stata 16 was used to perform all analyses and p-value 0.05 was deemed significant.

The hypothetical idea for the study was to assess the association between TP and PV and the analytical procedure can be found in S1 Fig.

### Ethical requirements

All DHS surveys have been reviewed and approved by the ICF Institutional Review Board (IRB). Ethical procedures in the overall process of the survey, including coordination of activities, were strictly followed. Data were collected after taking informed consent, and all information was kept confidential. Country-specific DHS survey protocols are reviewed by the ICF IRB and typically by an IRB in the host country. ICF IRB ensures the right of human subjects surveys which comply with the U.S. Department of Health and Human Services regulations for the protection of human subjects (45 CFR 46), while the host country IRB ensures that the survey complies with laws and norms of the nation [48]. The legitimacy to use DHS data was obtained from MEASURE DHS. The data underlying the results presented in the study are available upon request. The data is not in the public domain and acquisition of the data can be obtained from DHS https://dhsprogram.com/data/dataset_admin/login_main.cfm.

## Results

The analysis involved a total of 26055 teenage women aged 15–19 years across the five countries. The overall mean age was 16.9 years in all countries; however, the mean age is statistically different among the countries (F-test = 21.6, p-value<0.0001). The Rao-Scott test showed that there was a statistically significant association between all covariates and country understudied (p<0.05) except the HH wealth index (see Table 1).

**Table 1:** Household, community and individual characteristics among teenagers in the five African countries, evidence from country-specific recent DHS.

Covariate	All countries	Country of residence					Rao-Scott χ^2^
		Burkina-Faso	Kenya	Malawi	Nigeria	Tanzania	
	Weighted %	Weighted %	Weighted %	Weighted %	Weighted %	Weighted %	
**Household characteristics**							
**Age of HH head**	43.64(16.06)	43.93(12.11)	43.29(16.89)	43.03(11.11)	46.11(28.65)	45.69(23.52)	21.16***ɸ
**Age of HH head category**							8.16***
<30	20.7	17.4	21.3	23.8	13.9	15.5	
30–39	26.6	28.0	26.9	26.4	25.0	24.7	
40–49	19.7	21.5	19.4	18.2	22.9	23.4	
50–59	14.3	15.2	14.7	13.2	15.6	15.2	
60–59	10.5	10.5	10.1	10.1	12.7	11.6	
70+	8.2	7.4	7.6	8.3	9.9	9.6	
**Sex of HH**							40.07***
Male	72.4	76.9	66.5	70.7	82.3	76.4	
Female	27.6	23.1	33.5	29.3	17.7	23.6	
**HH has Telephone**							17.85***
No	98.8	99.0	99.6	98.2	99.3	99.5	
Yes	1.2	1.0	0.4	1.8	0.7	0.5	
**Wealth index**							**1.71**
Poorest	19.6	18.6	20.3	19.4	21.2	19.2	
Poorer	19.2	19.3	17.7	19.4	21.3	18.2	
Middle	19.1	19.7	18.3	18.7	20.9	19.7	
Richer	20.2	19.4	22.2	19.6	19.5	21.4	
Richest	21.9	23.0	21.5	22.9	17.1	21.5	
**Number of HH members**							59.83***
<4	20.9	20.3	17.6	23.2	22.0	14.7	
4–5	23.0	14.7	28.0	25.1	22.1	19.6	
6–8	36.6	30.4	36.9	40.4	30.6	37.6	
9+	19.5	34.6	17.5	11.3	25.3	28.1	
**HH has electricity**							111.91***
No	74.7	65.6	68.4	86.5	47.8	78.3	
Yes	25.3	34.4	31.6	13.5	52.2	21.7	
**HH has radio**							85.73***
No	48.3	45.6	34.9	56.6	42.7	49.1	
Yes	51.7	54.4	65.1	43.4	57.3	50.9	
**HH has color TV**							103.05***
No	76.4	71.3	67.7	86.3	56.6	80.8	
Yes	23.6	28.7	32.3	13.7	43.4	19.2	
**Where HH food is prepared**							218.30***
In the HH	25.1	36.6	45.9	7.6	37.5	35.3	
Separate HH	45.3	12.7	46.8	60.4	30.6	44.9	
Outdoors	29.4	49.7	7.3	31.9	31.8	19.7	
Other	0.2	1.0	0.0	0.1	0.1	0.1	
**HH has mobile phone**							249.32***
No	29.0	17.3	17.0	42.8	14.8	22.1	
Yes	71.0	82.7	83.0	57.2	85.2	77.9	
**HH has watch**							258.97***
No	79.6	72.3	81.2	89.0	46.7	77.9	
Yes	20.4	27.7	18.8	11.0	53.3	22.1	
**HH own land for agriculture**							56.23***
No	33.1	45.8	38.1	24.5	37.5	37.3	
Yes	66.9	54.2	61.9	75.5	62.5	62.7	
**HH number animal own**							50.27***
None	47.2	54.8	33.5	49.3	52.5	43.2	
1+animal	52.8	45.2	66.5	50.7	47.5	56.8	
**Relationship structure**							23.01***
No+1 adult	20.7	19.6	29.1	17.9	19.8	16.5	
Two adults, opp sex	38.6	36.6	33.1	42.0	37.6	39.0	
Two adults, same sex	5.1	4.6	6.5	5.0	4.0	3.1	
Three+ related adults	31.8	34.5	26.5	31.9	37.3	32.6	
Unrelated adults	3.9	4.7	4.8	3.2	1.3	8.8	
**Place of residence**							
Urban	27.4	33.5	31.9	17.4	45.1	37.3	31.89***
Rural	72.6	66.5	68.1	82.6	54.9	62.7	
**Individual characteristics**							
**Individual age**	16.94(1.43)	16.95(1.13)	16.96(1.53)	16.94(0.98)	16.85(2.54)	16.98(2.22)	5.07***ɸ
**Individual age category**							**1.16**
15–17	60.5	61.4	60.3	60.0	62.1	58.6	
18–19	39.5	38.6	39.7	40.0	37.9	41.4	
**Educational level**							355.26***
None	15.8	55.9	2.3	2.6	25.8	6.0	
Primary	49.8	21.4	49.9	70.4	10.4	58.9	
Secondary	33.4	22.6	45.0	26.6	61.1	35.0	
Higher	1.0	0.1	2.8	0.4	2.7	0.1	
**Marital status**							32.37***
Never married	75.3	67.6	86.8	73.2	76.6	74.7	
Married	22.6	31.5	11.9	23.5	22.8	23.0	
Divorced	2.0	0.8	1.2	3.1	0.5	2.3	
Widowed	0.1	0.1	0.1	0.2	0.1	0.0	
**Knowledge of pregnancy**							253.96***
No knowledge	82.1	100.0	100.0	71.0	60.4	80.9	
Have knowledge	17.9	0.0	0.0	29.0	39.6	19.1	
**Knows modern contraceptive**							65.09***
Knows no method	7.4	8.7	3.6	6.4	18.5	5.8	
Knows method	92.6	91.3	96.4	93.6	81.5	94.2	
**Family planning awareness**							114.96***
Not aware	60.8	47.1	66.4	63.8	75.8	37.1	
Aware	39.2	52.9	33.6	36.2	24.2	62.9	
**Currently abstaining**							41.86***
No	93.3	91.7	97.2	91.6	96.8	92.4	
Yes	6.7	8.3	2.8	8.4	3.2	7.6	
**Currently working**							279.05***
No	61.7	36.1	91.2	60.0	64.4	55.1	
Yes	38.3	63.9	8.8	40.0	35.6	44.9	

NOTE: Rao-Scott is a Design-based Chi-square test;

ɸ is F-test estimates from analysis of variance;

HH = Household;

TV = Television;

P-value indication:

* = p-value<0.05,

** = p-value<0.01,

*** = p-value<0.001.

### Prevalence of teenage pregnancy among adolescent women aged 15–19 years

The overall prevalence of TP among women aged 15–19 years was 25.4% (95%CI = 24.4–26.4) with the highest prevalence occurring among Malawians [29.0% (95%CI = 27.4–30.7)]. The prevalence of ever experience of PV among teenagers across the five countries was 24.2% (95%CI = 22.3–26.2). The highest prevalence of PV was recorded among Nigerians with a prevalence rate of 31.8% (95%CI = 28.5–35.3) (see S4 Table).

### Household and community factors influencing teenage pregnancy among adolescent women aged 15–19 years

Estimates from Poisson regression show that the HH characteristics significantly influencing TP among women aged 15–19 years in the five countries were the number of HH members. The prevalence ratio (PR) of TP decreases as the number of HH members increases. Within countries, Nigerians experienced higher PR compared with the remaining four countries. Place of residence as the only community characteristic was identified to influence TP in all countries. The significant rural-urban differential was observed to be disadvantageous among teenagers residing in rural communities. Overall, the PR of experiencing TP was 56% higher among rural teenagers compared with their urban peers [aPR(95%CI) = 1.56 (1.42–1.72)]. Within countries, rural residents in Nigeria were more than twice as likely to experience TP compared with urban residents [PR(95%CI) = 2.66(2.21–3.21)] (Table 2).

**Table 2:** Household and community characteristics influencing teenage pregnancy among the five countries, information from recent DHS.

HH and community characteristics	All countries	Country of residence				
		Burkina Faso	Kenya	Malawi	Nigeria	Tanzania
	aPR[95%CI]	aPR[95%CI]	aPR[95%CI]	aPR[95%CI]	aPR[95%CI]	aPR[95%CI]
**Age of HH head category**						
<30	**Ref**	**Ref**	**Ref**	**Ref**	**Ref**	**Ref**
30–39	1.09[0.98–1.19]	1.00[0.79–1.25]	1.07[0.87–1.33]	1.15[1.00–1.32]	1.05[0.89–1.23]	1.17[0.91–1.49]
40–49	0.99[0.88–1.11]	1.01[0.81–1.25]	0.96[0.76–1.22]	1.02[0.84–1.24]	0.94[0.79–1.13]	1.10[0.84–1.44]
50–59	0.98[0.87–1.11]	1.00[0.76–1.28]	0.97[0.75–1.26]	1.04[0.86–1.26]	0.92[0.74–1.14]	0.99[0.77–1.28]
60–59	0.94[0.82–1.06]	0.82[0.61–1.11]	0.80[0.60–1.06]	1.06[0.87–1.28]	0.92[0.74–1.14]	1.03[0.77–1.37]
70+	0.93[0.80–1.08]	0.84[0.58–1.21]	0.83[0.60–1.13]	1.04[0.84–1.29]	0.91[0.74–1.12]	1.00[0.73–1.37]
**Sex of HH**						
Male	**Ref**	**Ref**	**Ref**	**Ref**	**Ref**	**Ref**
Female	1.00[0.92–1.10]	0.99[0.80–1.22]	0.97[0.82–1.15]	1.03[0.89–1.19]	0.95[0.81–1.12]	1.06[0.87–1.29]
**HH has Telephone**						
No	**Ref**	**Ref**	**Ref**	**Ref**	**Ref**	**Ref**
Yes	1.13[0.80–1.59]	0.68[0.25–1.83]	0.69[0.27–1.79]	1.16[0.77–1.73]	0.73[0.36–1.46]	1.79[0.73–4.42]
**Wealth index**						
Richest	**Ref**	**Ref**	**Ref**	**Ref**	**Ref**	**Ref**
Poorest	0.82[0.71–0.94]**	1.16[0.76–1.78]	1.10[0.69–1.75]	0.87[0.70–1.08]	1.16[0.84–1.61]	1.55[0.92–2.62]
Poorer	0.84[0.73–0.96]**	1.06[0.73–1.55]	0.91[0.57–1.43]	0.98[0.80–1.21]	1.13[0.84–1.51]	1.41[0.84–2.35]
Middle	0.88[0.77–1.00]*	1.16[0.82–1.63]	0.92[0.60–1.42]	1.04[0.85–1.26]	1.01[0.78–1.28]	1.11[0.67–1.82]
Richer	0.89[0.80–1.00]*	1.01[0.78–1.30]	1.02[0.74–1.39]	0.96[0.79–1.18]	0.99[0.81–1.22]	1.14[0.76–1.72]
**Number of HH members**						
9+	**Ref**	**Ref**	**Ref**	**Ref**	**Ref**	**Ref**
<4	3.03[2.70–3.35]***	5.25[4.25–6.49]***	1.92[1.50–2.46]***	2.38[1.96–2.88]***	4.82[3.97–5.85]***	1.88[1.57–2.25]***
4–5	1.23[1.09–1.39]***	1.92[1.45–2.54]***	1.00[0.78–1.26]	0.97[0.78–1.20]	2.25[1.82–2.77]***	0.83[0.66–1.05]
6–8	0.85[0.75–0.96]**	1.19[0.90–1.56]	0.72[0.57–0.91]**	0.66[0.53–0.82]***	1.34[1.08–1.67]**	0.75[0.63–0.89]***
**HH has electricity**						
Yes	**Ref**	**Ref**	**Ref**	**Ref**	**Ref**	**Ref**
No	1.02[0.93–1.14]	0.72[0.58–0.88]**	1.02[0.76–1.36]	1.06[0.86–1.31]	0.95[0.82–1.10]	1.07[0.74–1.53]
**HH has radio**						
Yes	**Ref**	**Ref**	**Ref**	**Ref**	**Ref**	**Ref**
No	1.03[0.95–1.11]	1.09[0.94–1.28]	1.02[0.87–1.22]	0.98[0.86–1.11]	1.02[0.92–1.13]	0.93[0.79–1.08]
**HH has color TV**						
yes	**Ref**	**Ref**	**Ref**	**Ref**	**Ref**	**Ref**
No	1.18[1.06–1.32]***	1.08[0.89–1.32]	1.02[0.76–1.38]	1.09[0.87–1.38]	1.08[0.92–1.28]	0.94[0.65–1.35]
**Where HH food is prepared**						
In the HH	**Ref**	**Ref**	**Ref**	**Ref**	**Ref**	**Ref**
Separate HH	1.09[1.00–1.18]*	0.90[0.71–1.14]	1.09[0.93–1.28]	1.10[0.89–1.35]	0.89[0.77–1.02]	1.12[0.95–1.32]
Outdoors + Others	1.13[1.03–1.23]**	1.06[0.91–1.24]	1.05[0.79–1.39]	1.14[0.91–1.43]	0.93[0.81–1.06]	1.27[1.04–1.56]*
**HH has a mobile phone**						
Yes	**Ref**	**Ref**	**Ref**	**Ref**	**Ref**	**Ref**
No	1.13[1.04–1.23]***	1.09[0.89–1.34]	1.03[0.83–1.29]	1.04[0.93–1.14]	1.16[1.02–1.32]*	1.08[0.90–1.29]
**HH has watch**						
Yes	**Ref**	**Ref**	**Ref**	**Ref**	**Ref**	**Ref**
No	1.01[0.92–1.09]	1.00[0.84–1.18]	0.95[0.77–1.16]	0.91[0.76–1.08]	1.00[0.89–1.11]	0.89[0.74–1.06]
**HH own land for agriculture**						
Yes	**Ref**	**Ref**	**Ref**	**Ref**	**Ref**	**Ref**
No	1.02[0.95–1.11]	1.25[1.04–1.50]*	1.13[0.94–1.34]	1.04[0.92–1.18]	0.95[0.84–1.07]	0.92[0.76–1.13]
**HH number animal own**						
None	**Ref**	**Ref**	**Ref**	**Ref**	**Ref**	**Ref**
1+animal	0.99[0.93–1.07]	1.18[0.99–1.40]	0.99[0.83–1.18]	0.96[0.86–1.06]	1.05[0.94–1.17]	0.88[0.74–1.06]
**Relationship structure**						
No+1 adult	**Ref**	**Ref**	**Ref**	**Ref**	**Ref**	**Ref**
Two adults, opposite sex	1.03[0.93–1.13]	0.95[0.73–1.22]	1.00[0.81–1.22]	0.95[0.81–1.11]	1.26[1.06–1.50]**	1.08[0.84–1.39]
Two adults, same sex	1.02[0.84–1.24]	0.94[0.62–1.42]	0.86[0.61–1.19]	1.01[0.75–1.35]	1.46[1.12–1.89]**	1.43[0.99–2.08]
Three+ related adults	1.06[0.95–1.18]	1.02[0.80–1.30]	1.01[0.81–1.26]	0.95[0.79–1.13]	1.25[1.05–1.48]*	1.30[1.02–1.65]*
Unrelated adults	0.94[0.77–1.15]	1.00[0.68–1.47]	0.93[0.64–1.37]	0.83[0.58–1.19]	1.14[0.72–1.80]	1.04[0.74–1.44]
**Place of residence**						
Urban	**Ref**	**Ref**	**Ref**	**Ref**	**Ref**	**Ref**
Rural	1.56[1.42–1.72]***	1.80[1.45–2.22]***	1.19[0.99–1.44]	1.25[1.06–1.46]**	2.66[2.21–3.21]***	1.60[1.29–1.98]***

NOTE: Ref: Reference category;

HH = Household; TV = Television;

P-value indication:

* = p-value<0.05,

** = p-value<0.01,

*** = p-value<0.001.

### Individual factors influencing teenage pregnancy among adolescent women aged 15–19 years

It was quite surprising to identify from Poisson estimates that all individual demographic characteristics studied influence TP in the five countries except working status. Increasing age was estimated to have an increased PR of experiencing TP. The prevalence of TP among older adolescents (18–19 years) was approximately two-thirds higher compared with young adolescents [aPR(95%CI) = 1.60[1.49–1.71)]. Intuitively, marital status was observed to have a high influence on TP among women aged 15–19 years. Surprisingly, married and divorced/widowed women in Nigeria were more than approximately 30 and 26 folds likely to experience TP compared with never-married teenagers [aPR(95%CI) = 29.98(23.91–37.60) and 25.35(18.70–34.37) respectively]. Counter-intuitively, abstaining from sex was observed to have a higher PR of TP compared with non-abstinence among older teenagers in all countries. This result was observed to have approximately 4-folds of TP among older teenagers in Kenya [aPR(95%CI) = 3.55(2.86–4.41)] (see Table 3).

**Table 3:** Individual characteristics influencing teenage pregnancy among five African low- and-middle income countries, information from recent DHS.

Individual characteristics		Country of residence				
	All countries	Burkina Faso	Kenya	Malawi	Nigeria	Tanzania
	aPR[95%CI]	aPR[95%CI]	aPR[95%CI]	aPR[95%CI]	aPR[95%CI]	aPR[95%CI]
**Age category**						
15–17	**Ref**	**Ref**	**Ref**	**Ref**	**Ref**	**Ref**
18–19	1.60[1.49–1.71]***	1.74[1.48–2.02]***	1.78[1.52–2.09]***	1.45[1.30–1.61]***	1.51[1.39–1.63]***	1.90[1.63–2.21]***
**Educational level**						
Secondary + Higher	**Ref**	**Ref**	**Ref**	**Ref**	**Ref**	**Ref**
None	1.09[1.00–1.19]*	1.60[1.21–2.09]***	0.94[0.75–1.19]	1.10[0.90–1.33]	1.03[1.95–1.12]	1.64[1.26–2.12]***
Primary	1.30[1.19–1.41]***	1.75[1.30–2.36]***	1.34[1.16–1.55]***	1.13[0.99–1.27]	1.04[0.93–1.15]	1.57[1.26–1.94]***
**Marital status**						
Never married	**Ref**	**Ref**	**Ref**	**Ref**	**Ref**	**Ref**
Married	7.28[6.51–8.13]***	13.08[9.41–18.17]***	7.02[5.95–8.26]***	6.09[5.14–7.21]***	29.98[23.91–37.60]***	4.31[3.59–5.16]***
Divorced + Widowed	6.76[5.85–7.80]***	11.37[6.87–18.83]***	7.21[5.59–9.30]***	5.63[4.62–6.88]***	25.35[18.70–34.37]***	3.82[2.95–4.94]***
**Knowledge of pregnancy**						
No knowledge	**Ref**	**-**	-	**Ref**	**Ref**	**Ref**
Have knowledge	1.25[1.18–1.33]***			1.29[1.19–1.40]***	1.13[1.05–1.21]***	1.39[1.23–1.58]***
**Knows modern contraceptive**						
Knows no method	**Ref**	**Ref**	**Ref**	**Ref**	**Ref**	**Ref**
Knows method	1.78[1.53–2.05]***	1.27[0.98–1.62]	2.53[1.70–3.77]***	3.08[1.80–5.25]***	1.19[1.08–1.31]***	2.38[1.35–4.18]**
**Family planning awareness**						
Aware	**Ref**	**Ref**	**Ref**	**Ref**	**Ref**	**Ref**
Not Aware	1.02[0.97–1.07]	0.93[0.85–1.02]	1.08[0.95–1.22]	1.05[0.97–1.13]	1.01[0.94–1.09]	0.87[0.78–1.02]
**Currently abstaining**						
No	**Ref**	**Ref**	**Ref**	**Ref**	**Ref**	**Ref**
Yes	2.23[2.10–2.41]***	2.07[1.87–2.29]***	3.55[2.86–4.41]***	2.24[2.02–2.48]***	1.76[1.62–1.90]***	2.77[2.41–3.19]***
**Currently working**						
No	**Ref**	**Ref**	**Ref**	**Ref**	**Ref**	**Ref**
Yes	0.99[0.94–1.04]	1.05[0.95–1.17]	1.19[1.01–1.39]*	1.05[0.96–1.14]	1.03[0.96–1.09]	1.17[1.04–1.32]**

NOTE: P-value indication:

* = p-value<0.05,

** = p-value<0.01,

*** = p-value<0.001.

### Experience of physical violence among adolescent pregnant women aged 15–19 years

TP correlation with PV was assessed to establish the relationship. The results show that there exists a positive significant correlation between TP and experience of PV (both raw scores and dummy outcome) (for detailed correlation coefficient values see S5 Table). Unexpectedly, segregating PV by TP status, the analysis showed that, almost all adolescents with TP have ever experienced any form PV in all the countries (p-value<0.001) (see S6 Table).

In all countries, TP was statistically associated with PV and that, PV among adolescent women who were pregnant was approximately 5-folds significant compared with those who were not pregnant [aPR(95%CI = 4.70 (3.86–5.73)]. For the country-specific experience of physical violence, TP was highly associated with PV [Bukina Faso: aPR(95%CI = 4.29(3.47–5.30), Kenya: aPR(95%CI = 3.21(2.67–3.85), Malawi: aPR(95%CI = 3.67(2.97–4.52), Nigeria: aPR(95%CI = 3.39(2.85–4.03), and Tanzania: aPR(95%CI = 4.72(3.86–5.77)] (see Table 4).

**Table 4:** Modified poisson estimation of the experience of domestic physical violence among teenage pregnant women in five African low- and middle-income countries, information from recent DHS.

Demographic characteristics	All countries	Country of residence				
		Burkina Faso	Kenya	Malawi	Nigeria	Tanzania
	aPR[95%CI]	aPR[95%CI]	aPR[95%CI]	aPR[95%CI]	aPR[95%CI]	aPR[95%CI]
**Teenage pregnancy**						
No	**Ref**	**Ref**	**Ref**	**Ref**	**Ref**	**Ref**
Yes	4.70[3.86–5.73]***	4.29[3.47–5.30]***	3.21[2.67–3.85]***	3.67[2.97–4.52]***	3.39[2.85–4.03]***	4.72[3.86–5.77]***
**Age group**						
15–17	**Ref**	**Ref**	**Ref**	**Ref**	**Ref**	**Ref**
18–19	0.95[0.84–1.06]	1.11[0.94–1.31]	0.90[0.70–1.15]	1.05[0.90–1.23]	1.09[0.94–1.26]	0.95[0.84–1.06]
**Educational level**						
Higher	**Ref**	**Ref**	**Ref**	**Ref**	**Ref**	**Ref**
None	1.07[0.86–1.32]	0.88[0.71–1.09]	0.92[0.67–1.27]	0.86[0.68–1.09]	0.93[0.80–1.07]	1.07[0.86–1.34]
Primary	1.08[0.90–1.30]	1.07[0.83–1.38]	1.01[0.81–1.25]	0.94[0.78–1.12]	1.29[1.06–1.57]**	1.09[0.90–1.31]
**Marital status**						
Never married	**Ref**	**Ref**	**Ref**	**Ref**	**Ref**	**Ref**
Married	1.21[1.07–1.36]	0.94[0.76–1.17]	1.09[0.92–1.28]	1.23[1.09–1.40]***	0.80[0.67–0.95]**	1.21[1.07–1.37]**
DSW	1.35[1.11–1.65]	1.35[0.89–2.04]	1.08[0.86–1.30]	1.25[1.02–1.54]*	0.92[0.66–1.29]	1.35[1.11–1.65]**
**Knowledge on pregnancy**						
No knowledge	**Ref**			**Ref**	**Ref**	**Ref**
Have knowledge	0.92[0.85–1.00]			0.92[0.82–1.02]	0.95[0.83–1.07]	0.92[0.85–1.01]
**Know contraceptive**						
Knows no methods	**Ref**	**Ref**	**Ref**	**Ref**	**Ref**	**Ref**
Knows methods	1.36[0.86–2.16]	1.06[0.76–1.43]	1.62[0.71–3.70]	2.06[1.13–3.74]*	1.32[1.09–1.62]**	1.37[0.85–2.18]

NOTE: P-value indication:

* = p-value<0.05,

** = p-value<0.01,

*** = p-value<0.001.

## Discussion

This study uses the recent DHS data sets of five LMICs in Africa to obtain the prevalence of teenage pregnancy among women aged 15–19 years and the factors that are associated with it. Overall, one in four teenage women aged 15–19 years were pregnant, of which rural residence was identified to significantly influence the likelihood of an adolescent becoming pregnant in all countries studied. Overall, the log-likelihood of an adolescent becoming pregnant was 56% higher among rural teenagers compared with their urban peers. Elsewhere outside of Africa, one in five teenage women aged 15–19 years have been pregnant, with more than 64% of such pregnancies unplanned [49]. Sexual abuse, exploitation were significant associated factors identified elsewhere. However, in Africa, a recent systematic review identified low self-esteem, curiosity, cell phone usage, and substance abuse as individual determinants of adolescent pregnancy [50]. A host of socio-cultural, environmental, economic as well as health-related factors contribute to the problem of adolescent pregnancy [50]. Interestingly, the number of HH members was found to have a decreasing TP log-likelihood ratio as the number of HH increases. TP has been established to be more common in the context of significant changes in family life including family size in these recent times [51]. The probable reason for this finding could that, lager family size, of course, older adults could serve as caretakers to the younger ones.

Studies have shown that up to 90% of teenage pregnancy occurs among married teenagers where intimate partner violence (IPV) is common [1,36]. We found out that, teenage women aged 15–19 years in Nigeria, who were either married or widowed, were more than 20 times more likely to experience violence, compared to teenage women who were never married. The probable reason could be that, in Africa, premarital pregnancy is considered a shame-based culture [52] and in other to avoid shame, these pregnant women get married when they heard about the pregnancy. This suggests that tackling the problem of teenage pregnancy and intimate partner violence among teenage women requires a closer look at early marriages. The problem of early marriages is very common in Africa. However, they remain an under-researched human right violation, that seeks to silence and constrain the spaces within which women can speak [53,54]. Most women who marry early are unemployed children who are susceptible to violence and abuse, often truncating the developmental potential of such children [55]. Early marriages are deep-rooted in the social, cultural, and religious fabrics of communities that make eradication very difficult. However, universal female education and female child empowerment may produce sustainable results.

Abstaining from sex was observed to have a high PR of pregnancy among teenagers aged 15–19 years compared with non-abstaining teenagers in all countries. For example, in Kenya, abstaining teenage women were more than 3.5 times more likely to have experienced teenage pregnancy compared with girls who are not abstaining from sex. This result, at a cursory look, seems impossible because trials have demonstrated the effectiveness of abstinence-centered health education intervention among adolescent girls [56–58]. However, the theoretically, fully protective abstinence intentions often fail among African adolescent girls [59,60] in the light of developmental pressure and other factors confronting teenagers in the communities in which they live such as cultural silence surrounding sexual matters, poor parental supervision or neglect within large and ‘broken’ families, deficient parent-child communication, inadequate neighborhood safety, poverty, inattention to (or lack of access to alternative support for) teenagers who may be sexually abused by family members and limited access to adolescent-friendly services among others. This results in teenagers claiming to be abstaining, whereas in practice they may occasionally fail to abstain. This makes such theoretical ‘abstainers’ unprotected as they are less likely to adopt a contraceptive method.

Approximately, one in four adolescents aged 15–19 years experienced any form of PV. Interestingly, almost all adolescent pregnant women in all countries have ever experienced any form of PV. Intimate partner violence (IPV) among all adolescents from a review of facility-based studies in Africa reported prevalence as high as 40% [1,36]. The difference in the high value of 40% by [36] compared to findings in the present study of 24.2% may be due to the difference in study settings as the former was a facility-based study, where victims of violence were more likely to present at a health facility with complaints, thereby overestimating the prevalence in the community. Physical violence among pregnant women is not without consequence for mother and child.

TP among women who have ever experience PV was over four times compared to those who have never experience PV in all countries. It was imperative to establish that, among all countries, physical violence is highly associated with TP. This makes PV an influencing factor for teenage pregnancy among women aged 15–19 years.

## Limitation

This study has some significant limitations that must not be overlooked. Firstly, the vast variation in years of the most current DHS data sets available for use of concern. The data set used spanned a period of 8 years with Burkina Faso’s current DHS conducted in 2010, while that of Nigeria was conducted in 2018. Kenya conducted its latest DHS in 2014 while both Malawi’s and Tanzania’s current DHS were conducted in 2015/2016. Secondly, datasets from only five African LMICs were used which would make generalizations of the findings to the whole of Africa problematic. Attempts were made at correcting this. However, we were faced with challenges as some countries did not have datasets that included violence which was the primary outcome of this analysis. Also, the presence of physical violence as considered in this study within the last 12 months could probably mean some of the adolescents were not yet pregnant. Although the results were derived from a robust analysis of nationally representative datasets which is a strength in itself, the authors advise in the light of the aforementioned that the results be contextualized appropriately when making inferences. Although all authors are from Ghana, DHS data from Ghana was not included because experience of physical violence was not captured.

## Conclusion

There is a high prevalence of pregnancy and PV among adolescent women aged 15–19 years. About a quarter of teenage women ever experienced physical violence. Counter-intuitively, pregnant teenage women were at a higher disadvantage to ever experience physical violence. We recommend gender-sensitive approaches in addressing the issue of Sustainable Development Goal (SGD) 5.2 (*Eliminate all forms of violence against all women and girls in the public and private spheres*, *including trafficking and sexual and other types of exploitation*) among teenage women to have better lives.

## Supporting information

S1 Fig Analysis framework defining factors influencing teenage pregnancy and the likelihood of experiencing physical violence among teenage pregnant women in five low-and-middle-income countries. (DOCX)

S1 Table Primary, secondary and covariates variables. (DOCX)

S2 Table Reliability test for experienced domestic physical violence. (DOCX)

S3 Table Sample and overall population among women aged 15–19 years. (DOCX)

S4 Table Prevalence of teenage pregnancy and experience of physical violence among women aged 15–19 years, estimates from all country current DHS. (DOCX)

S5 Table Significant pairwise correlations between teenage pregnancy and experience of physical violence on raw counts and binary outcomes within normalized domestic violence weight. (DOCX)

S6 Table Experience of physical violence prevalence by teenage pregnancy among women aged 15–19 years, estimates from all country current DHS. (DOCX)

## Abbreviations

CI: Confidence Interval
DHS: Demographic Health Survey
LMIC: Low Middle Income Country
PR: Prevalence ratio
PV: Physical Violence
SDG’s: Sustainable Development Goals
TP: Teenage pregnancy
