# Supplementary material for: Teenage pregnancy and experience of physical violence among women aged 15-19 years in five African countries: Analysis of complex survey data
Source: PLoS One. 2020 Oct 27;15(10):e0241348. doi: 10.1371/journal.pone.0241348 (PMC7591093; doi:10.1371/journal.pone.0241348)
Supplement: S1 Fig — (DOCX) [file pone.0241348.s001.docx]

 S2 Fig 1: Analysis framework defining the association between teenage pregnancy and ever experienced physical violence among teenage pregnant women in five low-and-middle-income countries
