# Supplementary material for: Teenage pregnancy and experience of physical violence among women aged 15-19 years in five African countries: Analysis of complex survey data
Source: PLoS One. 2020 Oct 27;15(10):e0241348. doi: 10.1371/journal.pone.0241348 (PMC7591093; doi:10.1371/journal.pone.0241348)
Supplement: S1 Table — (DOCX) [file pone.0241348.s002.docx]

S1 Table 1: Primary, secondary and covariates variables

| **Variable** | **Type of variable** | **Description** | **Measurement** | **Scale of measurement** | **Number of observations/Missing** |
| --- | --- | --- | --- | --- | --- |
| Physical violence | Outcome variable | Women 15-19 who have experienced physical violence during the 12 months preceding the survey were assessed by DHS | Raw scores recoded into Never experienced and Ever experienced | Discrete  Binary | 26055/0 |
| Teenage pregnancy | Outcome variable | Women aged 15-19 who have begun childbearing; either they have had a live birth or are pregnant with their first child | Yes or No | Binary | 26055/0 |
| Age of household head | Explanatory variable | Participant household head as at the time of data collection | Raw ages recoded into <30, 30-39, 40-49, 50-59, 60-59 and 70+ | Discrete  Categorical | 25108/947 |
| Sex of household head | Explanatory variable | Sex definition of household head | Male or Female | Binary | 25123/932 |
| Household has Telephone | Explanatory variable | Whether participant household has telephone (land-line) | No or Yes | Binary | 25111/944 |
| Wealth index | Explanatory variable | Wealth of the household relative to linear index of asset ownership | Poorest, poorer, middle, richer and richest | Ordinal | 25123/932 |
| Number of household members | Explanatory variable | Counts of individual household members | Recoded into <4, 4-5, 6-8 and 9+ | Discrete  Categorical | 26055/0 |
| Household has electricity | Explanatory variable | Whether participant household has electricity | No or Yes | Binary | 25117/938 |
| Household has radio | Explanatory variable | Whether participant household has radio | No or Yes | Binary | 25120/935 |
| Household has color television | Explanatory variable | Whether participant household has color television | No or Yes | Binary | 25116/939 |
| Where household food is prepared | Explanatory variable | Whether household food cooked in the house/separate/building/ outdoors | In the household, separate household and others | Categorical | 24807/1248 |
| Household has mobile phone | Explanatory variable | Whether participant household has mobile phone | No or Yes | Binary | 25119/936 |
| Household has watch | Explanatory variable | Whether participant household has watch | No or Yes | Binary | 25119/936 |
| Household own land for agriculture | Explanatory variable | Whether participant household own land for agriculture | No or Yes | Binary | 25123/932 |
| Household number of animals own | Explanatory variable | Number of animal’s household owns | Recoded into None or 1+animal | Binary | 26055/0 |
| Relationship structure | Explanatory variable | Participant relationship with household | None+1 adult; two adults, opposite sex; two adults, same sex; three+ related adults and unrelated adults | Categorical | 25123/932 |
| Place of residence | Explanatory variable | Participant type of place of residence | Urban or Rural | Binary | 26055/00 |
| Age of participant | Explanatory variable | Participant individual age as at the time of data collection | Raw ages of participant recoded into 15-17 and 1819 | Discrete  Categorical | 26055/0 |
| Educational level | Explanatory variable | Educational level of participant | None, primary, secondary or higher | Categorical | 26053/2 |
| Marital status | Explanatory variable | Participant marital status | Never married, married, divorced/widowed | Categorical | 26055/0777 |
| Knowledge on pregnancy | Explanatory variable | Participants were asked “can women get pregnant after birth and before period” with yes or no answer | No knowledge and have knowledge | Binary | 26055 |
| Knows modern contraceptive | Explanatory variable | Whether participant knows any conceptive method like; folkloric, traditional and/or modern methods | Knows no method and knows method | Binary | 26055 |
| Family planning awareness | Explanatory variable | Whether participant has heard about family planning on radio, television, newspaper/magazine, from peers or text messages on mobile phone last few months | Recoded as not aware and aware | Binary | 26055 |
| Currently abstaining | Explanatory variable | Whether participant is currently abstaining from sex at the data collection | Yes or no | Binary | 26055 |
| Currently working | Explanatory variable | Whether participant is currently working as at the data collection | Yes or no | Binary | 26055 |
