# Supplementary material for: Teenage pregnancy and experience of physical violence among women aged 15-19 years in five African countries: Analysis of complex survey data
Source: PLoS One. 2020 Oct 27;15(10):e0241348. doi: 10.1371/journal.pone.0241348 (PMC7591093; doi:10.1371/journal.pone.0241348)
Supplement: S2 Table — (DOCX) [file pone.0241348.s003.docx]

S1 Table 2: Reliability test for experienced domestic physical violence

| **Item** | **NE** | **EE** | **Alpha** |
| --- | --- | --- | --- |
|  | **Weighted %** | **Weighted %** |  |
| Ever been pushed, shook or had something thrown by husband/partner | 95.8 | 4.2 | 0.69 |
| Ever been slapped by husband/partner | 89.1 | 10.9 | 0.71 |
| Ever been punched with fist or hit by something harmful by husband/partner | 95.7 | 4.3 | 0.70 |
| Ever been kicked or dragged by husband/partner | 96.4 | 3.6 | 0.70 |
| Ever been strangled or burnt by husband/partner | 99.4 | 0.6 | 0.77 |
| Ever been threatened with knife/gun or other weapon by husband/partner | 99.1 | 0.9 | 0.74 |
| Ever CS physical violence by husband/partner | 100.0 | 0.0 | - |
| Ever had arm twisted or hair pulled by husband/partner | 96.0 | 4.0 | 0.75 |
| No person other than husband/partner ever physically hurt respondent | 79.8 | 20.2 | 0.80 |
| Frequency of being hit in last 12 months by other than husband/partner | 46.2 | 53.8 | 0.76 |
| Respondent was not hurt by anyone during a pregnancy | 96.0 | 4.0 | 0.74 |
| Previous husband: ever hit, slap, kick, or physically hurt respondent | 79.2 | 20.8 | 0.74 |
| **Test of reliability** |  |  | 0.76 |

NOTE: NE is Never experienced physical violence and EE is Ever experienced physical violence
