# Supplementary material for: Teenage pregnancy and experience of physical violence among women aged 15-19 years in five African countries: Analysis of complex survey data
Source: PLoS One. 2020 Oct 27;15(10):e0241348. doi: 10.1371/journal.pone.0241348 (PMC7591093; doi:10.1371/journal.pone.0241348)
Supplement: S3 Table — (DOCX) [file pone.0241348.s004.docx]

S1 Table 3: Sample and overall population among women aged 15-19 years

| **Country** | **DHS women aged 15-49 years population** | **UN estimated women aged 15-49 years population** |
| --- | --- | --- |
| Burkina Faso | 17087 | 7124048 |
| Kenya | 31079 | 21952628 |
| Malawi | 24562 | 7727531 |
| Nigeria | 41821 | 91379196 |
| Tanzania | 13266 | 23375 |
