# Supplementary material for: Teenage pregnancy and experience of physical violence among women aged 15-19 years in five African countries: Analysis of complex survey data
Source: PLoS One. 2020 Oct 27;15(10):e0241348. doi: 10.1371/journal.pone.0241348 (PMC7591093; doi:10.1371/journal.pone.0241348)
Supplement: S4 Table — (DOCX) [file pone.0241348.s005.docx]

S1 Table 4: Prevalence of teenage pregnancy and experience of physical violence among women aged 15-19 years, estimates from all country current DHS

| **Country** | **Teenage pregnancy** | **Experience of physical violence** |
| --- | --- | --- |
|  | **Prevalence[95%CI]** | **Prevalence[95%CI]** |
| Burkina Faso | 23.6[21.7-25.7] | 20.5[17.8-23.6] |
| Kenya | 18.1[16.7-19.6] | 31.6[27.0-36.5] |
| Malawi | 29.0[27.4-30.7] | 23.0[19.7-26.7] |
| Nigeria | 18.7[17.2-20.4] | 31.8[28.5-35.3] |
| Tanzania | 26.7[24.3-29.1] | 21.9[19.3-24.7] |
| All countries | 25.4[24.4-26.4] | 24.2[22.3-26.2] |
