# Supplementary material for: Teenage pregnancy and experience of physical violence among women aged 15-19 years in five African countries: Analysis of complex survey data
Source: PLoS One. 2020 Oct 27;15(10):e0241348. doi: 10.1371/journal.pone.0241348 (PMC7591093; doi:10.1371/journal.pone.0241348)
Supplement: S5 Table — (DOCX) [file pone.0241348.s006.docx]

S1 Table 5: Significant pairwise correlations between teenage pregnancy and experience of physical violence on raw counts and binary outcomes within normalized domestic violence weight

| **Primary outcome** | **Secondary outcome** | **P-value** |
| --- | --- | --- |
| Experience of physical violence-Raw scores | 0.19 | <0.0001 |
| Experience of physical violence-Binary | 0.21 | <0.0001 |
