# Supplementary material for: Teenage pregnancy and experience of physical violence among women aged 15-19 years in five African countries: Analysis of complex survey data
Source: PLoS One. 2020 Oct 27;15(10):e0241348. doi: 10.1371/journal.pone.0241348 (PMC7591093; doi:10.1371/journal.pone.0241348)
Supplement: S6 Table — (DOCX) [file pone.0241348.s007.docx]

S1 Table 6: Experience of physical violence prevalence by teenage pregnancy among women aged 15-19 years, estimates from all country current DHS

| **Teenage pregnancy** | **All countries** | **Country** | | | | |
| --- | --- | --- | --- | --- | --- | --- |
|  |  | **Burkina Faso** | **Kenya** | **Malawi** | **Nigeria** | **Tanzania** |
|  | %[95%CI] | %[95%CI] | %[95%CI] | %[95%CI] | %[95%CI] | %[95%CI] |
| No | 18.4[15.6-21.5] | 23.9[20.5-27.6] | 30.3[25.5-35.5] | 22.3[18.5-26.6] | 33.9[30.0-38.0] | 18.3[15.5-21.5] |
| Yes | 99.1[97.1-99.8] | 99.0[97.9-99.5] | 98.7[94.9-99.6] | 98.5[96.0-99.5] | 98.3[96.1-99.2] | 99.1[97.1-99.7] |
| Rao-Scott χ^2^ | 582.9*** | 1158.5*** | 246.9*** | 402.9*** | 402.8*** | 570.1*** |

NOTE: Weighted percentages estimates; P-value indication: ***=p-value<0.0001
